# Minimal Efficacy of Single-Agent Anti-PD-(L)1 Re-Exposure in Anti-PD-(L)1-Refractory Merkel Cell Carcinoma: A Retrospective Cohort Study of 16 Patients

**DOI:** 10.3390/cancers18162673

**Published:** 2026-08-18

**Authors:** Peter Y. Ch’en, Yuzheng Zhang, Daniel S. Hippe, Rashmi Bhakuni, Tomoko Akaike, Evan T. Hall, Paul Nghiem

**Affiliations:** 1 Department of Dermatology, University of Washington, 850 Republican Street, Box 358050, Seattle, WA 98109, USA; peterc99@uw.edu (P.Y.C.);; 2 Clinical Research Division, Fred Hutch Cancer Center, Seattle, WA 98109, USA

**Keywords:** immune checkpoint inhibitor, immunotherapy, neuroendocrine and adrenal tumor, radiotherapy/radioimmunotherapy, relapse, re-exposure, rechallenge

## Abstract

**Background/Objectives:** Anti-PD-(L)1 immune checkpoint inhibitors (ICIs) provide durable responses in nearly 50% of patients with advanced Merkel cell carcinoma (MCC). However, for those progressing on first-line ICI therapy, optimal subsequent therapy remains unclear. Although presumed to have limited benefit, the efficacy of re-exposure with anti-PD-(L)1 alone has not been formally reported. We assessed real-world outcomes of patients within a Seattle-based MCC repository who received this salvage approach. **Methods:** Among 106 patients who received salvage therapy after first-line ICI progression, 16 underwent single-agent anti-PD-(L)1 monotherapy re-exposure during salvage. Patients progressing >3 months after their last immunotherapy dose were excluded. Outcomes included progression-free survival (PFS), disease-specific survival (DSS), and objective response rate (ORR). **Results:** The median time from end of first-line ICI therapy to anti-PD-(L)1 re-exposure was 51 days (IQR 22–92). Most patients switched between PD-1 and PD-L1 inhibitors (n = 9), while others were re-exposed with the same agent (n = 5) or a different PD-1 inhibitor (n = 2). One of 16 patients experienced a partial response with the same PD-1 inhibitor (ORR 6%; 95% CI: 0.2–30%) at 3 months after re-exposure, followed by progression 10 months after re-exposure. Median PFS was 2.2 months (95% CI: 1.3–5.1 months), and median DSS was 14.7 months (95% CI: 10.4–NR). **Conclusions:** These data suggest that re-exposure with anti-PD-(L)1 monotherapy confers minimal and short-lived benefit in ICI-refractory MCC, reinforcing the need to develop alternative salvage strategies. Future trials for ICI-refractory MCC mandating an ICI monotherapy arm are unlikely to be appealing to patients or physicians based on a low chance of clinical benefit for this approach.

## Simple Summary

Immunotherapy with PD-1 pathway blockade is the standard initial therapy for advanced Merkel cell carcinoma (MCC), an aggressive skin cancer. Unfortunately, over half of patients with advanced MCC develop resistance to immunotherapy. This study investigated whether restarting the same class of immunotherapy (“re-exposure”) is effective for these patients. We found that this approach provided minimal benefit: only one of 16 patients experienced tumor shrinkage, lasting 10 months before their tumors progressed. These findings indicate that reusing the same class of drugs is unlikely to benefit patients once immunotherapy resistance develops. Therefore, patients with immunotherapy-resistant MCC likely require alternative strategies, such as radiation, combination immunotherapy, or clinical trials of novel agents. Importantly, clinical trials in this setting should likely avoid an anti-PD-(L)1 monotherapy re-exposure arm as it appears unlikely to provide meaningful clinical benefit or to represent a compelling option for patients or treating clinicians.

## 1. Introduction

Merkel cell carcinoma (MCC) is an uncommon and aggressive skin cancer associated with exposure to extensive ultraviolet light, advanced age, and immunosuppression. The incidence of MCC is approximately 3200 new cases per year in the United States and rising [1,2,3,4]. In the United States, around 80% of MCCs are induced by the Merkel cell polyomavirus (MCPyV), and the remaining 20% arise exclusively from mutations caused by ultraviolet radiation [5,6,7]. Approximately 40% of MCC patients experience disease recurrence, and patients with advanced disease typically require systemic therapy [8].

Prior to 2017, cytotoxic chemotherapy was the first-line treatment for advanced MCC. Though chemotherapy induces a response in about 60% of patients, durability is typically short-lived [9,10]. Only ~5% of patients experience continued benefit from chemotherapy at 1–2 years [11].

In 2017, the first anti-programmed death-ligand 1 (anti-PD-L1) immune checkpoint inhibitor (ICI), avelumab, was approved for use in the United States by the FDA, followed by the approvals of pembrolizumab (anti-PD-1 in 2018) and retifanlimab (anti-PD-1 in 2023) [12]. Anti-PD-(L)1 ICI therapy quickly overtook chemotherapy as the preferred first-line treatment for advanced MCC, with durable responses in ~50% of patients with MCC at 2 years [13]. Since the introduction of ICIs, there has been a >2-fold increase in survival at the population level for advanced MCC [14].

However, for the 50% who progress on first-line ICI therapy, multidisciplinary input for salvage strategies is essential because there is no clear second-line option for treatment once ICI resistance develops [15]. Salvage options often involve a combination of an ICI (e.g., addition of a CTLA-4 inhibitor) and radiation or surgery based on the disease progression pattern. However, enrollment in a clinical trial, if feasible, is often preferred in this setting [16]. Trials for ICI-refractory MCC often test whether a novel agent can augment an anti-PD-(L)1 agent (e.g., the MATRiX trial testing an ATR inhibitor ± avelumab; NCT05947500) [17].

Although presumed to have limited benefit, the efficacy of re-exposure with anti-PD-(L)1 monotherapy has not been formally reported for patients with ICI-refractory MCC. In order to better contextualize outcomes from combination therapy trials in ICI-refractory disease, it is important to understand the potential benefit attributable to anti-PD-(L)1-ICI alone in this setting. Thus, we assessed real-world outcomes of ICI-refractory patients within a Seattle-based MCC repository who received this salvage approach.

## 2. Materials and Methods

In brief, this is a retrospective analysis of a Seattle-based MCC observational repository with an eligibility cutoff of 16 August 2024. The repository was re-queried on 13 May 2025 to update available follow-up and outcomes. This repository was approved by the Institutional Review Board at Fred Hutch Cancer Center (IRB #6585, Seattle, WA, USA). Eligible patients were those who had progressed during or after first-line ICI and received salvage therapy for MCC. Full original study criteria are available in the previously published study [18]. For the present study, the cohort consisted of a subset of patients who received anti-PD-(L)1 monotherapy re-exposure. These patients progressed either while receiving first-line anti-PD-(L)1 or within 12 weeks of their last dose [19,20].

Details of interventional treatments starting from initiation of first-line ICI to last follow-up date were captured. The characteristics of the treatments, including start and end dates and objective responses with corresponding dates, were extracted. Each eligible patient also had their electronic medical record manually reviewed for quality control and to construct the case descriptions included in this study.

### Statistical Analysis

Time 0 for all analyses was defined as the initiation date of anti-PD-(L)1 monotherapy re-exposure. Outcomes were progression-free survival (PFS), disease-specific survival (DSS), and objective response rate (ORR). Progression-free survival (PFS) was defined as the duration from initiation of ICI re-exposure until next PD or death from any cause. Disease-specific survival (DSS) was defined as the duration from initiation of ICI re-exposure until death from MCC. Attribution of death to MCC was determined using clinical records from Seattle and collaborating medical teams based on the treating physician’s determination of the cause of death. Cause of death was classified as due to MCC, non-MCC, or unknown. In the case of unknown cause of death, DSS was censored at the time of death (n = 3). Objective response rate (ORR) was defined as the fraction of patients who experienced an objective response (PR or CR) after initiation of ICI re-exposure before next PD or death divided by the total number of evaluable patients.

All statistical analyses were conducted using R version 4.5.0 (R Foundation for Statistical Computing, Vienna, Austria). PFS and DSS probabilities were estimated using the Kaplan–Meier estimator. Median PFS and DSS were estimated as the time corresponding to a 50% event-free probability on the Kaplan–Meier curve.

## 3. Results

**Figure 1:**
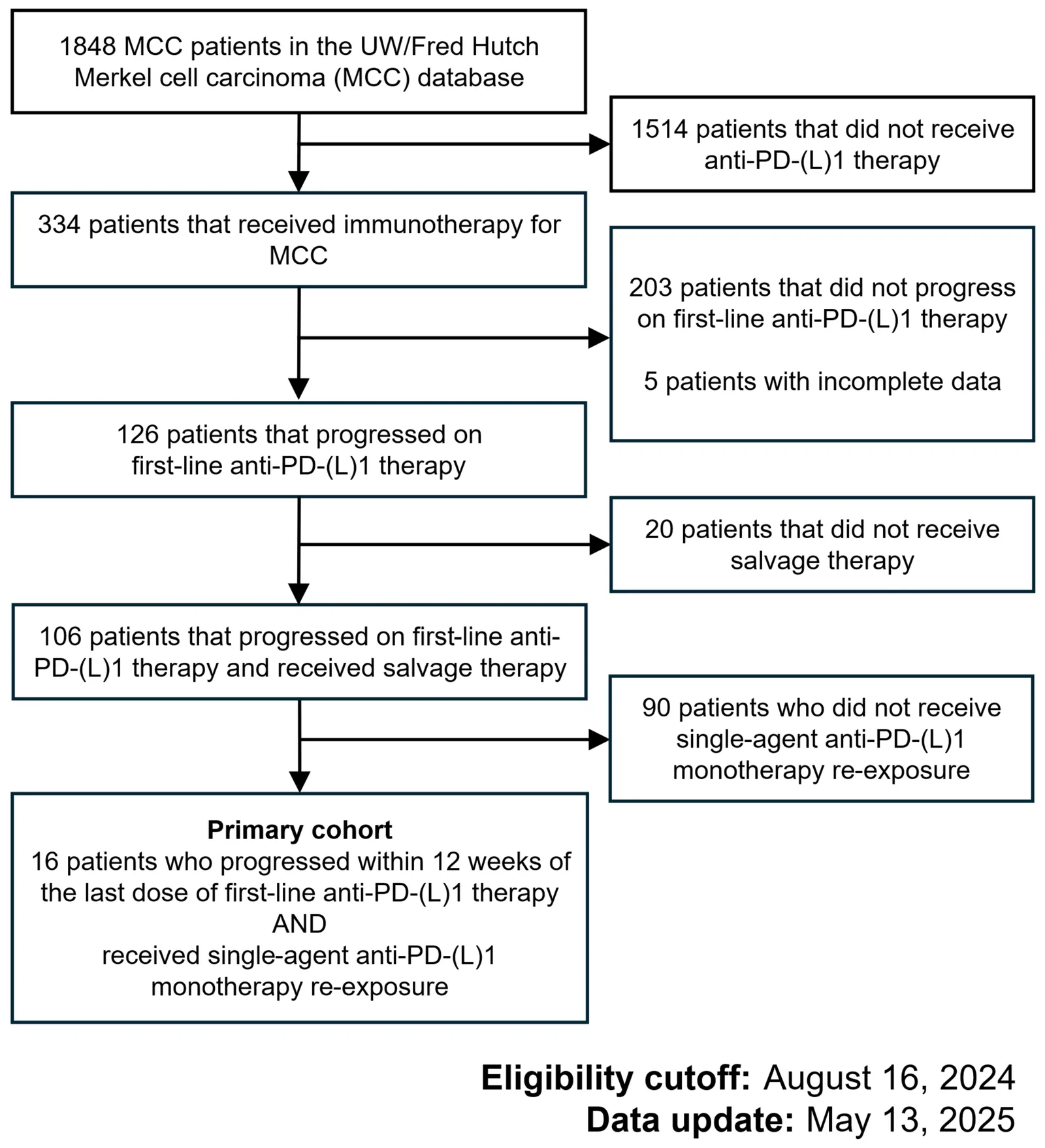
Flow chart of patient cohort selection.

At study initiation, there were 1848 MCC patients in the Seattle MCC registry, of which 334 patients received ICIs for MCC (Figure 1) [18]. From these 334 patients, 106 underwent at least one salvage therapy after progressing on first-line anti-PD-(L)1, thus meeting the original study’s inclusion criteria [18].

Among these 106 patients, 16 had progressed within 12 weeks of their last dose of first-line ICI therapy and received single-agent anti-PD-(L)1 re-exposure during their salvage therapy course. This subset was not separately analyzed in the initial study [18]. The median time to re-exposure after progression on first-line anti-PD-(L)1 ICI was 51 days (IQR 22–92).

**Figure 2:**
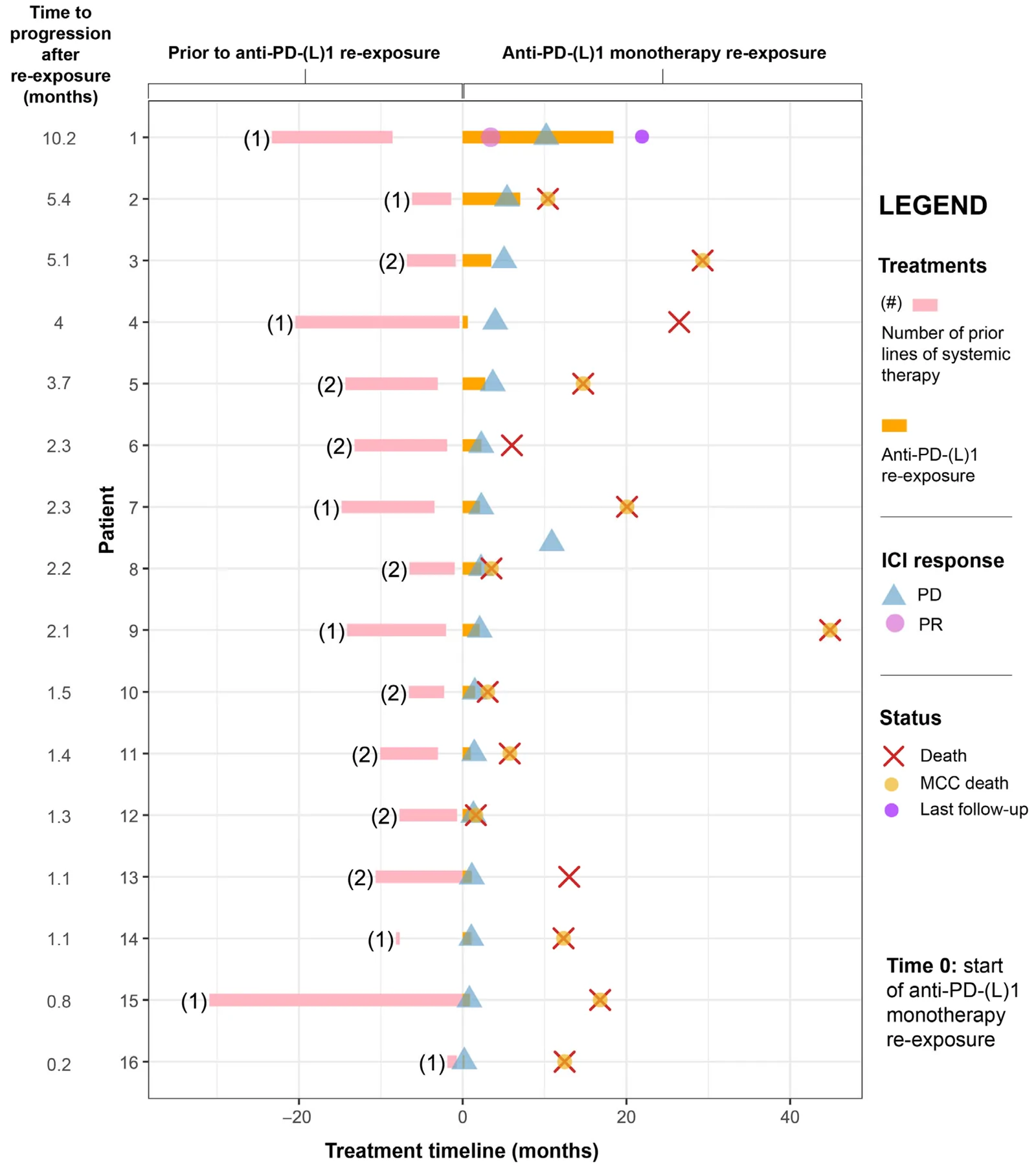
Swimmer plot of 16 patients who received anti-PD-(L)1 ICI monotherapy re-exposure following progression on anti-PD-(L)1 ICI, ordered by duration of benefit and time to progression.

A swimmer plot summarizing the number of systemic treatments received preceding re-exposure and the anti-PD-(L)1 re-exposure course of these patients are depicted in Figure 2. A corresponding per-patient summary is available in Table 1.

**Figure 3:**
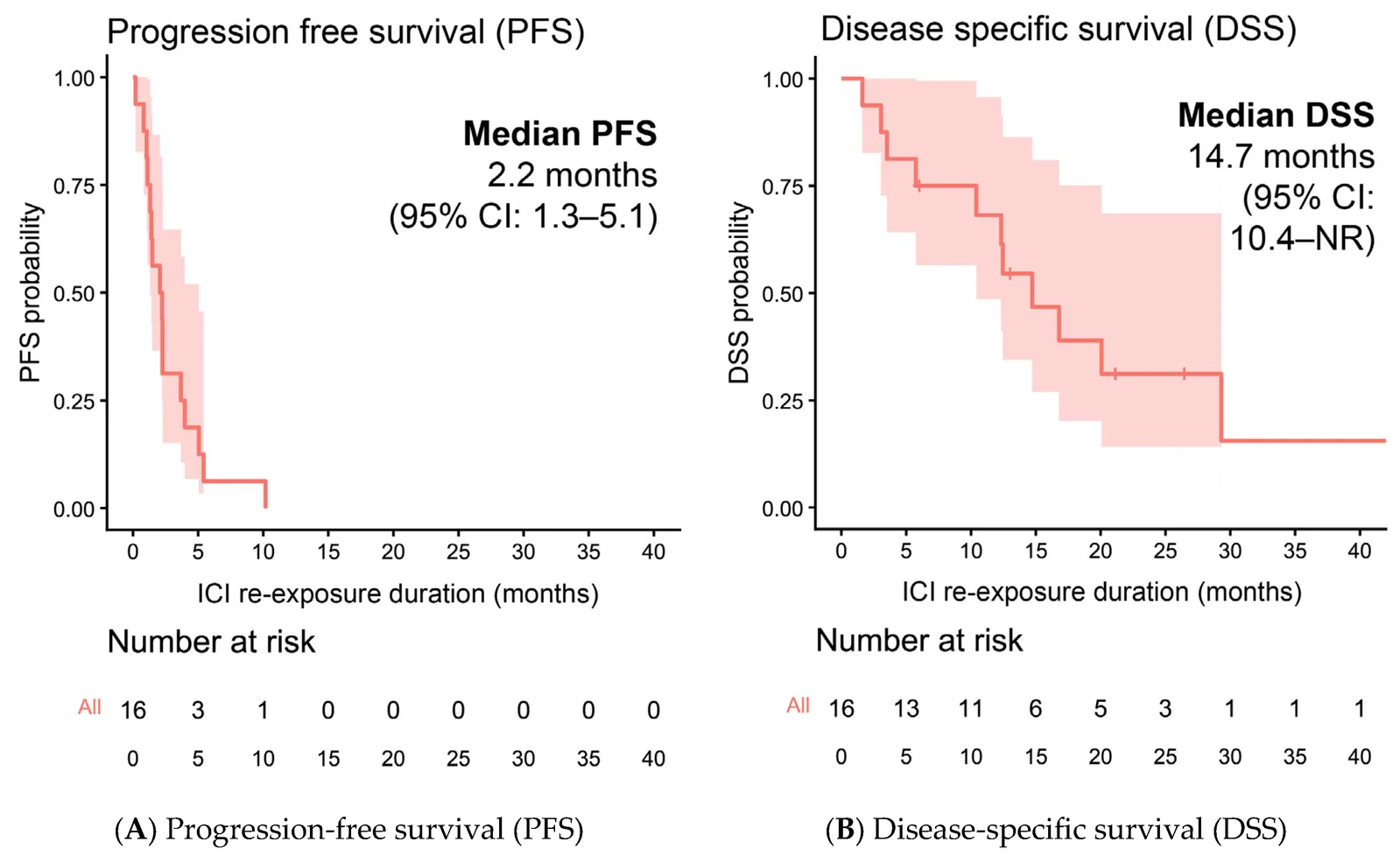
Survival outcomes of patients receiving anti-PD-(L)1 re-exposure, time 0: re-exposure start. (**A**) Progression-free survival (PFS), (**B**) disease-specific survival (DSS). Solid lines indicate Kaplan–Meier survival estimates, and shaded bands indicate 95% confidence intervals.

Median PFS was 2.2 months (95% CI: 1.3–5.1), and median DSS was 14.7 months (95% CI: 10.4–NR) (Figure 3). Median follow-up time from ICI re-exposure initiation was 12.5 months (IQR 5–21). Most patients switched between PD-1 and PD-L1 inhibitors (6 PD-L1 to PD-1; 3 PD-1 to PD-L1); 5 were re-exposed to the same agent and 2 switched to a different PD-1 inhibitor.

One of 16 patients in this study experienced an objective response (ORR 6%, 95% CI: 0.2–30%; partial response with same PD-1 inhibitor) to single-agent anti-PD-(L)1 re-exposure, with progression 10 months after re-exposure. This case is described below.

### Patient #1 (Responder)

A man in his 60s who presented with stage IV MCC involving the thyroid and left inguinal and pelvic lymph nodes experienced a complete radiographic response after 7 months of first-line pembrolizumab. His course was complicated by immune-related inflammatory arthritis, which was well controlled with prednisone 10 mg daily. At month 11, while still receiving pembrolizumab and low-dose prednisone, his disease recurred in the thyroid, pelvic lymph nodes, and, newly, the perirenal region. Because this occurred during concurrent immunosuppression rather than with fully intact antitumor immunity, his ICI-refractory designation should be interpreted with caution. Pembrolizumab was discontinued at month 12 with the development of immune-related pancreatitis, managed with high-dose prednisone (100 mg daily, tapered) and mycophenolate mofetil (1 g twice daily).

Pembrolizumab was restarted at month 20 (261 days after discontinuation), defining time 0 for re-exposure; immunosuppression had been de-escalated to prednisone 7.5 mg daily beforehand. The patient experienced a partial response at 3 months of re-exposure (month 23), but the disease progressed approximately 10 months into re-exposure (month 30). Pembrolizumab was continued while local salvage was pursued, including radiation to bilateral renal metastases (25 Gy in 5 fractions). Recurrent immune-related arthritis of the same and new joints required low-dose prednisone, methotrexate, and tocilizumab. Pembrolizumab was discontinued after approximately 18 months of re-exposure (month 38) due to a combination of disease progression and treatment-related adverse events.

## 4. Discussion

For patients with ICI-refractory MCC, there remains a large unmet need for effective second-line therapies following progression on anti-PD-(L)1 agents [18,21,22,23,24,25,26,27]. Multiple ongoing clinical trials aim to address this gap; however, many incorporate continued anti-PD-(L)1 therapy as part of the treatment backbone [17,28,29]. In this setting, it is important to delineate the contribution of the prior ICI, on which the patient has already progressed, versus the added investigational therapy. Careful interpretation is required to ensure that any observed clinical benefit is attributable to the novel agent or combination strategy, rather than residual or confounding effects of continued ICI therapy. In this cohort, outcomes were dismal for patients who underwent a single-agent anti-PD-(L)1 monotherapy re-exposure salvage strategy. Only 1 of 16 patients experienced a transient ~7-month partial response upon anti-PD-(L)1-only re-exposure, suggesting that this monotherapy salvage strategy confers minimal benefit in the ICI-refractory setting. It also suggests that this approach is not an attractive or feasible trial arm for randomization in future MCC trial designs.

ICI re-exposure is a rational strategy for “late progressors” (when progression occurs >3 months after cessation of therapy), but the logic is weaker when disease progresses while the drug is still present [18,19,20,30,31]. Anti-PD-1 antibodies can persist on T cells for over two months; thus, progression within this window more likely reflects true ICI resistance rather than a late progression [32,33,34]. Since the literature defines ICI resistance inconsistently, and including late progressors may inflate apparent re-exposure response rates, we required documented progression within 12 weeks of the last dose of PD-1 pathway blockade for inclusion.

ICI re-exposure has been previously evaluated across multiple solid tumors, with overall modest efficacy outside of select clinical contexts. A systematic review including 3579 patients reported a pooled ORR of 18.4%, with higher response rates observed among patients who previously benefited from ICIs and those with longer initial treatment durations, suggesting that retained immune sensitivity is a key predictor of response [35]. Consistent with this, retrospective data in metastatic renal-cell carcinoma (RCC) demonstrated an ORR of 23% with re-exposure, with responses enriched among prior responders but still observed in a minority of patients overall [36]. Importantly, these ICI re-exposure studies included cases where the ICI re-exposure mechanism was different (e.g., CTLA-4 instead of PD-1 inhibition). In MCC, the addition of a CTLA-4 inhibitor to anti-PD-(L)1 therapy has been associated with an ORR of ~30% [37]. Therefore, we believe that these numbers would be even lower had these analyses been limited to the same mechanistic class of ICIs as in our study.

Prospective evidence further limits enthusiasm for routine anti-PD-(L)1 re-exposure following progression. In the phase 3 CONTACT-03 trial, continuation of PD-(L)1 blockade with atezolizumab in combination with cabozantinib did not improve progression-free or overall survival and increased toxicity compared to cabozantinib monotherapy in patients with RCC [38]. In contrast, higher response rates have been reported in melanoma patients re-exposed after prior disease control rather than progression (ORR 55%) [39]. Collectively, these data indicate that anti-PD-(L)1 ICI monotherapy re-exposure has limited benefit in ICI-refractory disease, supporting the need for alternative salvage approaches.

The sole responder in our cohort highlights the complexity of defining and managing ICI-refractory MCC in the setting of significant immune-related toxicity and concurrent immunosuppression [40]. Although the patient technically met criteria for ICI-refractory disease at the time of progression, this occurred while also receiving systemic immunosuppressive therapy for immune-related adverse events, which may have attenuated antitumor immune activity. Re-exposure with pembrolizumab resulted in a meaningful but transient partial response for ~7 months, suggesting that some degree of immune sensitivity was retained despite prior progression. However, the eventual disease progression and repeated immune-related adverse events in this patient spotlight the limited durability of benefit with ICI re-exposure in this context. Consistent with broader experience in ICI-refractory MCC, effective disease control ultimately required a multimodal salvage approach incorporating continued immunotherapy alongside localized treatments such as radiation, reflecting the need for multidisciplinary salvage strategies in this challenging population. As noted in prior studies, a combination of radiation with an ICI as salvage has been associated with survival benefit [18,41].

### Limitations of This Study

There are several limitations to this study. Given this study’s retrospective design of a single-center observational database, it is subject to selection bias. As a high-volume specialty referral care center for Merkel cell carcinoma with ready access to specialized care, patients may experience a quicker turnaround and may have benefited from readily available multidisciplinary input; generalizing results beyond this setting should be done with caution. Such high-volume centers have been associated with improved survival outcomes in national database studies [42].

Furthermore, the small sample size of patients who underwent anti-PD-(L)1 monotherapy re-exposure (n = 16) limits statistical power. It also restricts the ability to perform subgroup analyses to identify clinical or treatment-related factors associated with response, such as prior duration of benefit from first-line ICI therapy or immune-related toxicity history. Therefore, the findings may not fully capture the heterogeneity in outcomes across different patient subsets.

## 5. Conclusions

These findings suggest that anti-PD-(L)1 monotherapy re-exposure after progression on first-line anti-PD-(L)1 therapy provides minimal clinical benefit in patients with ICI-refractory MCC, with responses being rare and generally short-lived. In this cohort, most patients experienced rapid disease progression despite retreatment, reinforcing the limited utility of this strategy once resistance to initial ICI therapy has developed. After progression on first-line ICI therapy, changing single agents within the same mechanistic class is not an adequate salvage strategy.

These results have implications for clinical practice and trial design. In the salvage setting, emphasis should shift toward alternative multidisciplinary approaches, including combination immunotherapy (e.g., addition of anti-CTLA-4), novel agents (e.g., ATR inhibition), and multimodal strategies (e.g., addition of radiation to ICI therapy). Additionally, future clinical trials in ICI-refractory MCC should likely avoid inclusion of an ICI monotherapy control arm, as the low likelihood of meaningful benefit may limit both patient and clinician enthusiasm for enrollment.

## Figures and Tables

**Table 1:** Summary table of 16 patients with MCC who received anti-PD-(L)1 re-exposure after progression either while receiving first-line ICI or within 12 weeks of progression from the last dose of first-line ICI.

Patient ID	Age/Sex at ICI Re-Exposure	First Anti-PD-(L)1 ICI Agent	First ICI Duration (Months)	First ICI Best Response	Time to Re-Exposure from Last Dose of First ICI (Days)	Anti-PD-(L)1 Mechanism Change?	ICI Re-Exposure Agent	ICI Re-Exposure Duration (Months)	ICI Re-Exposure Best Response
1	70M	Pembrolizumab	15	CR	261	PD-1 → PD-1 (same agent)	Pembrolizumab	18	PR: 7 months
2	76F	Pembrolizumab	4	PD	43	PD-1 → PD-L1	Avelumab	7	PD
3	76F	Pembrolizumab	2	PD	26	PD-1 → PD-1 (same agent)	Pembrolizumab	3	PD
4	59M	Avelumab	20	PR	12	PD-L1 → PD-1	Pembrolizumab	0, 1 dose	PD
5	73M	Pembrolizumab	9	PR	93	PD-1 → PD-1 (same target)	Nivolumab	2	PD
6	73F	Atezolizumab	6	SD	58	PD-L1 → PD-1	Pembrolizumab	2	PD
7	71F	Pembrolizumab	11	PD	105	PD-1 → PD-1 (same agent)	Pembrolizumab	2	PD
8	67M	Avelumab	0	PD	30	PD-L1 → PD-1	Nivolumab	2	PD
9	60F	Nivolumab	12	CR	62	PD-1 → PD-1 (same target)	Pembrolizumab	2	PD
10	83F	Avelumab	1	PD	69	PD-L1 → PD-1	Pembrolizumab	1	PD
11	71M	Avelumab	1	PD	92	PD-L1 → PD-1	Pembrolizumab	1	PD
12	71M	Pembrolizumab	2	PD	21	PD-1 → PD-1 (same agent)	Pembrolizumab	1	PD
13	62F	Avelumab	7	PD	0	PD-L1 → PD-L1	Avelumab	1	PD
14	23F	Nivolumab	0	PD	234	PD-1 → PD-L1	Avelumab	1	PD
15	73M	Pembrolizumab	31	PR	0	PD-1 → PD-L1	Avelumab	1	PD
16	46M	Avelumab	1	PD	22	PD-L1 → PD-1	Nivolumab	0, 1 dose	PD

M: male; F: female; CR: complete response; PR: partial response; SD: stable disease; PD: progressive disease; PD-1: programmed death-1; PD-L1: programmed death-ligand 1; →: first-line agent mechanism → re-exposure agent mechanism.

## Data Availability

The original contributions presented in this study are included in this article. Further inquiries can be directed to the corresponding author.
